# Ophthalmic Review on Neonatal Harlequin Ichthyosis

**DOI:** 10.7759/cureus.44320

**Published:** 2023-08-29

**Authors:** Boon Juan Yeoh, Selvaraja Nanthini

**Affiliations:** 1 Ophthalmology, University of Malaya, Kuala Lumpur, MYS; 2 Ophthalmology, Hospital Raja Permaisuri Bainun, Ipoh, MYS

**Keywords:** rare congenital anomaly, ichthyosis, plate-like hyperkeratosis, congenital ichthyosis, harlequin ichthyosis

## Abstract

Harlequin ichthyosis is a rare congenital autosomal recessive disorder that causes hyperkeratosis or plate-like keratosis. Hyperkeratosis affects both upper and lower eyelids and causes defective eyelids. Lagophthalmos and persistent dry eye will cause desiccation of the cornea, possibly leading to complications such as ectropion, cornea ulceration, corneal perforation, etc. Harlequin ichthyosis requires regular ocular review to prevent ocular complications. In this child, he was born with defective eyelids, but subsequent management prevented the complications mentioned. This is a case of harlequin ichthyosis in a neonate from an ophthalmological point of view.

## Introduction

Harlequin ichthyosis is a congenital autosomal recessive disorder [1-3]. It results in hard thickened skin. This disorder has a prevalence of 1:300000 per population [1]. This is a case of harlequin ichthyosis in a neonate in the periphery of Sabah state, Malaysia. The child was born in a district hospital with specialist care. The population in this area has poor education and has difficulty understanding the disorder and the required care of such patients. Understanding the disorder takes precedence in preventing complications and, therefore, the well-being of the child. Infections are the main complications, both systemic and ocular, because of the loss of a functional skin barrier. In a neonate, a simple infection may be fatal. This is a case that also highlights the efficacy of therapy and the improvement seen with the usage of isotretinoin.

## Case presentation

A 34-week premature baby boy was born with generalized hyperkeratosis, skin cracking, facial dysmorphism, hypoplastic digits in all four limbs, and bilateral upper lid ectropion.

**Figure 1:**
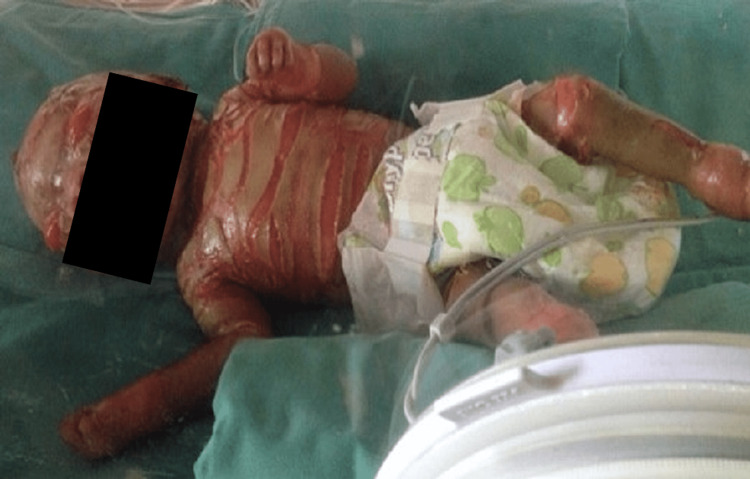
Patient at birth

Upon examination, the patient was active and well-hydrated. His upper eyelids revealed plate-like hyperkeratosis with ectropion, as shown in Figure 1. There were no exposed corneas seen. The bilateral conjunctivae were white, the corneas were clear, the pupils were round and reactive, and the fundus examination revealed a normal macula and normal optic disc, with no abnormalities seen. Systemic examination revealed intermittent thick, cracking skin sparing the joints and mobile areas.

**Figure 2:**
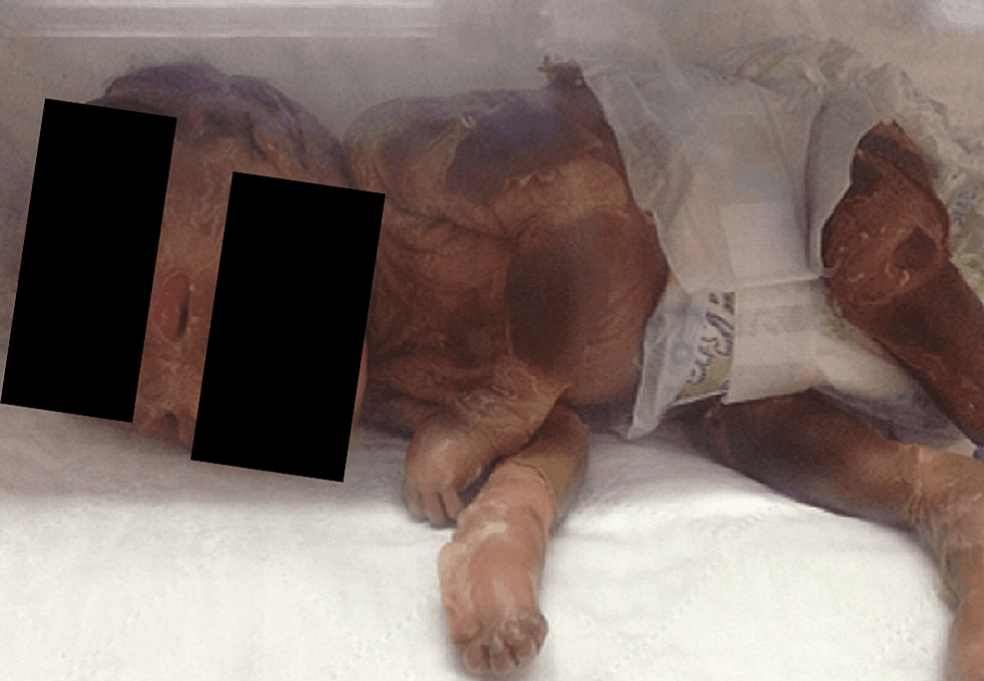
Patient at age one week Plate-like hyperkeratosis appears to be reducing, and parts of healthy are seen emerging at the eyelids

**Figure 3:**
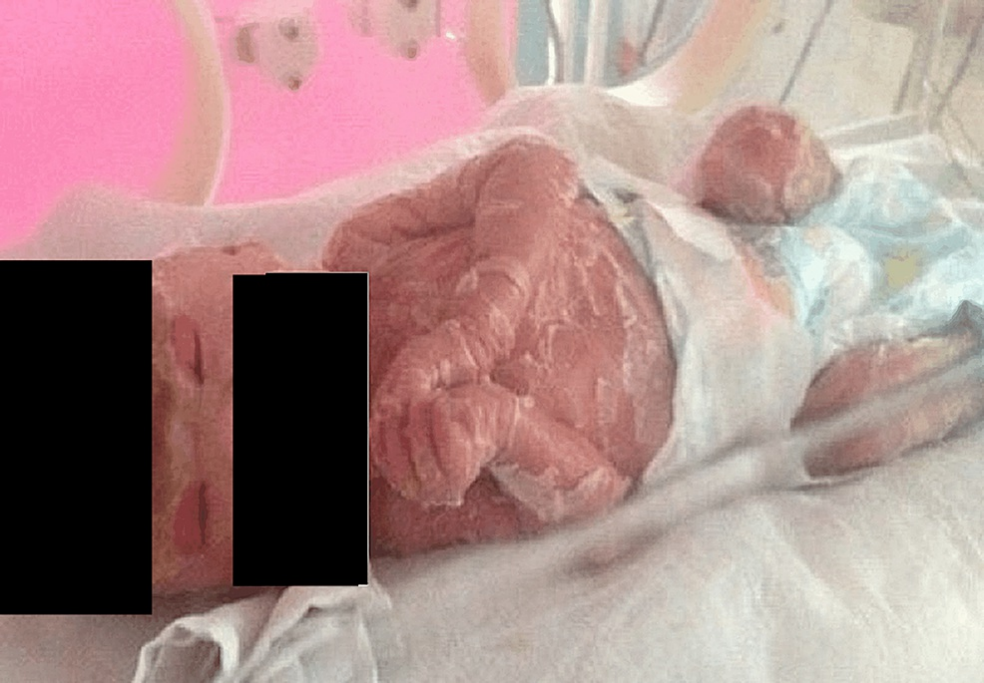
Patient at age two weeks Upper eyelids and lower eyelids reveal no lagophthalmos

A combined management plan was initiated with the pediatric team. The child was started on oral isotretinoin therapy and artificial tear drops to prevent exposure to keratitis. A lubricating ointment was also applied three times a day. The skin condition showed progressive involution during follow-up, as shown in Figures 2-3. However, in the third week of life, the child developed bilateral eye discharge. Swab culture and sensitivity results showed heavy growth of *Pseudomonas aeruginosa*. The child was treated with gentamicin eye drops four hourly for two weeks. The infection resolved after treatment and no complications occurred.

**Figure 4:**
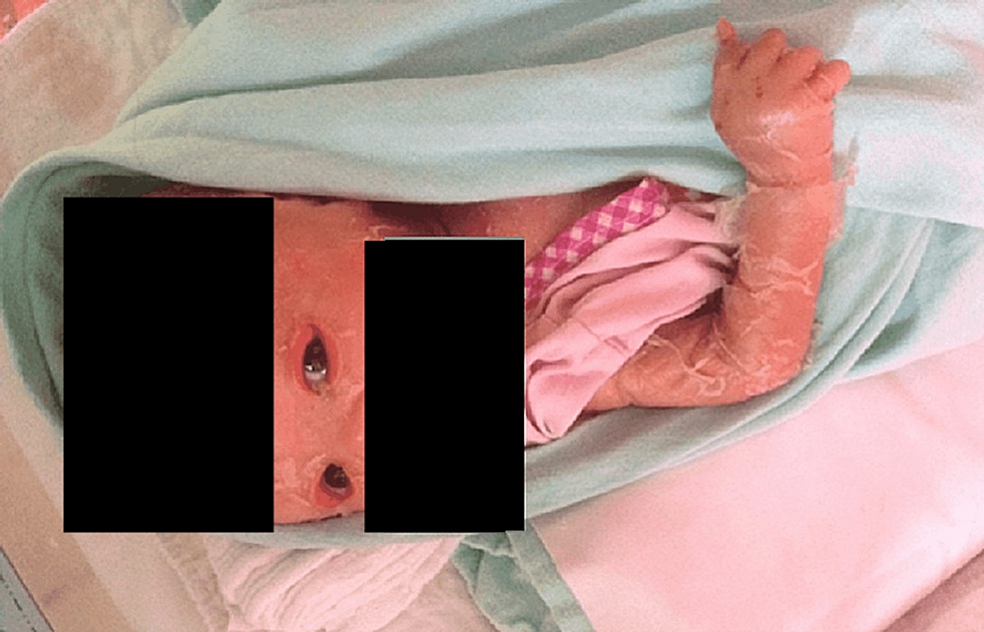
Patient at age two months old The patient is able to open his eyes spontaneously and focus on objects presented

By two months, the skin condition had improved significantly. Hyperkeratosis of the upper and lower eyelids resolved completely, as shown in Figure 4. Prevention of exposure keratopathy was successful. The child was discharged home on oral isotretinoin and artificial tear drops. However, the parents defaulted subsequent follow-up, and the child succumbed to death at four months old.

## Discussion

Ophthalmic management of the patient often requires the replacement therapy of tears to prevent dry eyes and complications. Ectropion correction has also been reported to be beneficial to these children [4]. In the event of an infection, aggressive antibiotic management is crucial to prevent further complications. The usage of isotretinoin is recommended to reduce keratosis [1-7]. Tarsorrhaphy was recommended for severe exposure keratopathy in some literature [4]. Non-syndromic patients with harlequin ichthyosis do not have other ocular abnormalities.

Oral isotretinoin has shown a remarkable result in this case. Delicate care is mandatory to moisturize and protect the skin and eyes [4]. The hard outer layer eventually peels off, leaving the vulnerable inner layers of the dermis exposed. In the past, the disorder was always fatal, whether due to dehydration or sepsis [8].

## Conclusions

As seen in this case, prevention of ocular complications is essential. Ocular infections are common due to the dysfunction of the primary barrier. As seen in this patient, ocular infections require treatment and review. These patients also require regular review of their eyelids, as eyelid abnormalities can lead to corneal injury, such as corneal exposure, abrasion, ulceration, and subsequent blindness. This patient was well cared for in the hospital and had healthy eyelids formed prior to discharge. However, the final care for the child ultimately falls to the parents. If the parents do not have a significant understanding of the patient's condition and do not continue to provide care, the survival of children with harlequin ichthyosis will be low.

## References

[1] (2023). Harlequin ichthyosis. https://rarediseases.org/rare-diseases/ichthyosis-harlequin-type/.

[2] Shibata A, Ogawa Y, Sugiura K, Muro Y, Abe R, Suzuki T, Akiyama M (2014). High survival rate of harlequin ichthyosis in Japan. J Am Acad Dermatol.

[3] Glick JB, Craiglow BG, Choate KA, Kato H, Fleming RE, Siegfried E, Glick SA (2017). Improved management of harlequin ichthyosis with advances in neonatal intensive care. Pediatrics.

[4] Al-Amry MA (2016). Ocular manifestation of ichthyosis. Saudi J Ophthalmol.

[5] Heap J, Judge M, Padmakumar B (2020). Harlequin ichthyosis from birth to 12 years. BMJ Case Rep.

[6] Akiyama M (2011). The roles of ABCA12 in keratinocyte differentiation and lipid barrier formation in the epidermis. Dermatoendocrinol.

[7] Katugampola RP, Finlay AY (2006). Oral retinoid therapy for disorders of keratinization: single-centre retrospective 25 years' experience on 23 patients. Br J Dermatol.

[8] Vahlquist A, Fischer J, Törmä H (2018). Inherited nonsyndromic ichthyoses: an update on pathophysiology, diagnosis and treatment. Am J Clin Dermatol.

